# RNA Expression of MMP12 Is Strongly Associated with Inflammatory Bowel Disease and Is Regulated by Metabolic Pathways in RAW 264.7 Macrophages

**DOI:** 10.3390/ijms25063167

**Published:** 2024-03-09

**Authors:** Laura Arosa, Miguel Camba-Gómez, Luis Francisco Lorenzo-Martín, Laura Clavaín, Miguel López, Javier Conde-Aranda

**Affiliations:** 1 Molecular and Cellular Gastroenterology Group, Health Research Institute of Santiago de Compostela (IDIS), 15706 Santiago de Compostela, Spain; laura.arosa.garcia@sergas.es (L.A.); miguel.camba.gomez@sergas.es (M.C.-G.); 2 Laboratory of Stem Cell Bioengineering, École Polytechnique Fédérale de Lausanne, 1015 Lausanne, Switzerland; luis.lorenzomartin@epfl.ch; 3 EGO Genomics, Scientific Park of the University of Salamanca, Adaja Street 4, Building M2, 37185 Villamayor, Spain; lclavain@egogenomics.com; 4 NeurObesity Group, Department of Physiology, Center for Research in Molecular Medicine and Chronic Diseases (CIMUS), University of Santiago de Compostela, 15782 Santiago de Compostela, Spain; m.lopez@usc.es; 5 CIBER Fisiopatologia de la Obesidad y Nutrición (CIBERobn), 15706 Santiago de Compostela, Spain

**Keywords:** metalloprotease, ulcerative colitis, macrophages, inflammation

## Abstract

Macrophage metalloelastase or matrix metalloproteinase-12 (MMP12) is a macrophage-specific proteolytic enzyme involved in the physiopathology of many inflammatory diseases, including inflammatory bowel disease. Although previously published data suggested that the modulation of MMP12 in macrophages could be a determinant for the development of intestinal inflammation, scarce information is available on the mechanisms underlying the regulation of MMP12 expression in those phagocytes. Therefore, in this study, we aimed to delineate the association of MMP12 with inflammatory bowel disease and the molecular events leading to the transcriptional control of this metalloproteinase. For that, we used publicly available transcriptional data. Also, we worked with the RAW 264.7 macrophage cell line for functional experiments. Our results showed a strong association of *MMP12* expression with the severity of inflammatory bowel disease and the response to relevant biological therapies. In vitro assays revealed that the inhibition of mechanistic target of rapamycin complex 1 (mTORC1) and the stimulation of the AMP-activated protein kinase (AMPK) signaling pathway potentiated the expression of *Mmp12*. Additionally, AMPK and mTOR required a functional downstream glycolytic pathway to fully engage with *Mmp12* expression. Finally, the pharmacological inhibition of MMP12 abolished the expression of the proinflammatory cytokine *Interleukin-6* (*Il6*) in macrophages. Overall, our findings provide a better understanding of the mechanistic regulation of MMP12 in macrophages and its relationship with inflammation.

## 1. Introduction

Inflammatory bowel disease (IBD) is a group of chronic and relapsing inflammatory disorders that generally include Crohn’s disease (CD) and ulcerative colitis (UC). Although the exact triggering cause of IBD is unknown, it is assumed to result from a complex interaction among the genetic background, environmental factors, dysbiosis of the microbiota, and a disrupted mucosal immune response [1]. In recent decades, the latter has been the subject of a plethora of research studies. In fact, most of the effective IBD therapies are designed to control several main routes involved in the dysregulated activation and infiltration of immune cells. However, these treatments present certain deficiencies, and more knowledge of the precise mechanisms controlling aberrant inflammatory responses is still necessary.

Matrix metalloproteinases (MMPs) are endopeptidases characterized by the presence of a zinc ion in their catalytic domain. The members of this family of proteins are generally subdivided based on differences in domain composition and substrate preference in collagenases, gelatinases, elastases, stromelysins, matrilysins, membrane-type MMPs, and other MMPs [2,3]. These are the main enzymes responsible for the remodeling of the extracellular matrix (ECM), although MMPs also play a major role in many other physiological and pathological processes, such as angiogenesis, wound healing, and inflammation [2,3,4]. Indeed, intestinal inflamed tissues frequently present altered MMP expression [5,6,7]. Moreover, these proteins can control the activity of relevant cytokines and chemokines [8,9,10], thereby impacting leukocyte migration during the development of intestinal inflammation [11,12].

Matrix metalloproteinase-12 (MMP12), which is also called macrophage metalloelastase, is a macrophage-specific proteolytic enzyme that degrades different substrates (elastin, laminin, type-IV collagen, fibronectin, and casein) [13]. The JAK/STAT, SOCS or PI3K signaling pathways regulate the expression of this enzyme [14,15,16], and in general, MMP12 is pivotal for macrophage functions, since macrophages from *Mmp12*^−/−^ mice presented impaired migratory capacity both in vitro and in vivo [17]. It is also noteworthy that MMP12 participates in different cardiovascular, skin, and respiratory inflammatory diseases and has emerged as an interesting putative therapeutic target for those conditions. Similarly, MMP12 expression was increased in the colonic mucosa and serum of IBD patients [18,19,20,21], and stool MMP12 protein levels showed high capacity for monitoring disease progression and predicting disease remission in pediatric patients with IBD [22], which suggested that MMP12, as observed for other inflammatory disorders, might play an active role in the development of intestinal inflammation. In fact, *Mmp12*^−/−^ mice displayed attenuated colitis severity in both acute and chronic DSS-induced colitis, and this was caused by a decrease in the epithelial permeability and, more importantly, by a reduction in the transmigration of macrophages through the intestinal epithelium [20].

The above data suggest that the modulation of MMP12 activity in macrophages could be a relevant therapeutic target for IBD and other inflammatory diseases. However, despite the current information, there are no data from an exhaustive analysis of colonic MMP12 levels in different cohorts of IBD patients. It is not known whether the master regulators of cell metabolism, AMP-activated protein kinase (AMPK) and mechanistic target of rapamycin (mTOR), can regulate MMP12 expression in macrophages. Therefore, in the current study, we explored several publicly available microarray and RNAseq datasets and used mouse RAW264.7 macrophages as an in vitro model to clarify those knowledge gaps. We found that *MMP12* is heavily associated with IBD disease activity and treatment response. Also, our results showed that *MMP12* expression in macrophages depends on the AMPK–mTOR axis, and the inhibition of that metalloproteinase downregulates the expression of the crucial pro-inflammatory mediator *Interleukin-6* (*Il6*). Collectively, our results contribute to a better characterization of the association of MMP12 with IBD and its mechanistic regulation in macrophages.

## 2. Results

### 2.1. MMP12 Is Strongly Associated with IBD and the Biological Therapeutic Response

**Figure 1:**
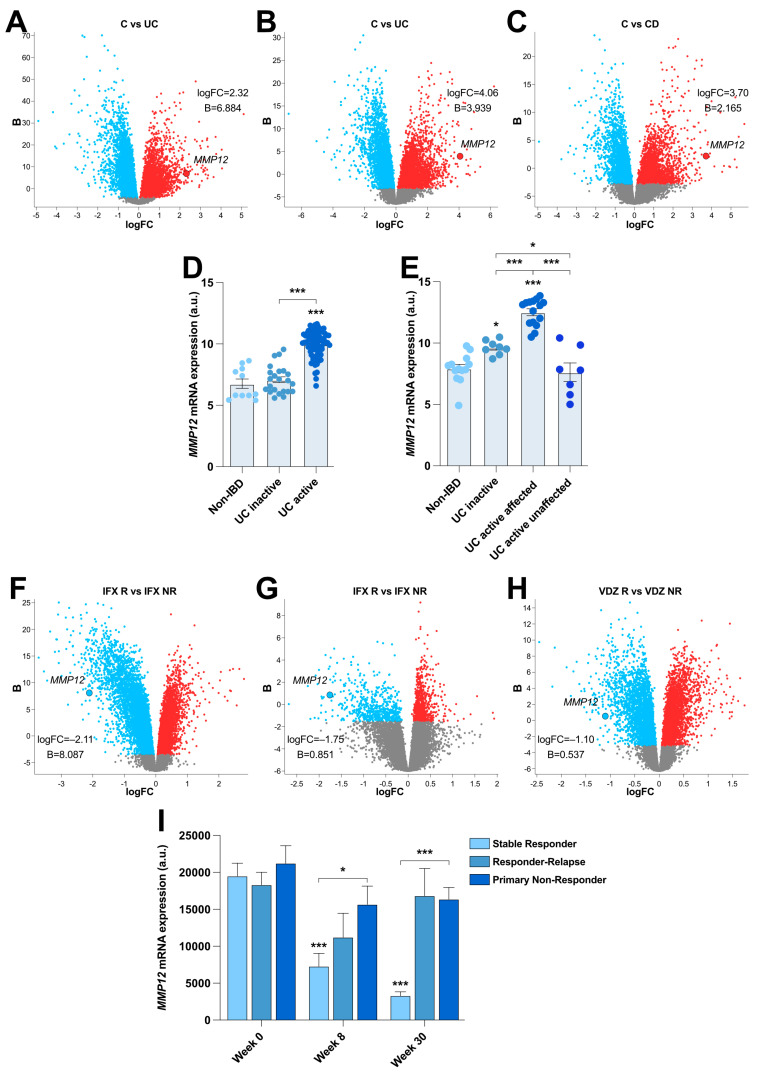
***Matrix metalloproteinase- 12*** (*MMP12*) expression is associated with inflammatory bowel disease (IBD) disease activity and the response to biological therapies. Volcano plots showing the differentially expressed genes (DEGs) in human colon biopsies of the GEO microarray datasets (**A**) GSE73661 (control; *n* = 12; ulcerative colitis (UC); *n* = 44) and (**B**,**C**) GSE16879 (control; *n* = 12; UC; *n* = 24; Crohn´s disease (CD); *n* = 37) in the indicated groups. The upregulated and downregulated transcripts are represented by red and blue dots, respectively. Relative *MMP12* expression in colon biopsies from non-IBD (*n* = 11), UC-inactive (*n* = 23), and UC-active (*n* = 74) patients from the GEO microarray dataset (**D**) GSE59071 and non-IBD (*n* = 13), UC-inactive (*n* = 8), affected UC-active (*n* = 15), and unaffected UC-active patients (*n* = 7) from the GEO microarray dataset (**E**) GSE38713. Volcano plots showing the DEGs in human colon biopsies of the GEO microarray datasets (**F**) GSE16879 (infliximab (IFX) R; *n* = 8; IFX NR; *n* = 16) and (**G**,**H**) GSE73661 (IFX R *n* = 8; IFX NR *n* = 15; vedolizumab (VDZ) R *n* = 8; VDZ NR *n* = 30) in the indicated groups. The upregulated and downregulated transcripts are represented by red and blue dots, respectively. (**I**) Relative *MMP12* expression in colon biopsies from the indicated groups of patients from the GEO microarray dataset GSE23597. B = log-odds; logFC = Fold Change. *, *p* ≤ 0.05; ***, *p* ≤ 0.001 relative to control, unless indicated differently.

First, we wanted to corroborate previous observations showing increased MMP12 levels in the colonic mucosa of patients with IBD. For this purpose, we focused on publicly available transcriptomic data, which confirmed the upregulation of colonic *MMP12* expression in two independent cohorts of patients with UC and one cohort of CD patients versus healthy controls (Figure 1A–C). Interestingly, we also found that *MMP12* expression was associated with clinical status, with *MMP12* transcripts being significantly increased in UC patients with active disease compared to those in inactive UC patients, in non-IBD controls (Figure 1D,E), and in unaffected tissue of active UC patients (Figure 1E). Similarly, *MMP12* mRNA expression was strongly decreased in infliximab (IFX) and vedolizumab (VDZ) responder UC patients relative to that in refractory patients of these two biological therapies (Figure 1F–H). In the case of anti-TNF therapy, this decrease remained significant in stable responders after thirty weeks of treatment, unlike that observed in non-stable responders or primary non-responders (Figure 1I).

### 2.2. MMP12 Correlates with an AMPK/mTOR Transcriptional Signature in UC Patients

**Figure 2:**
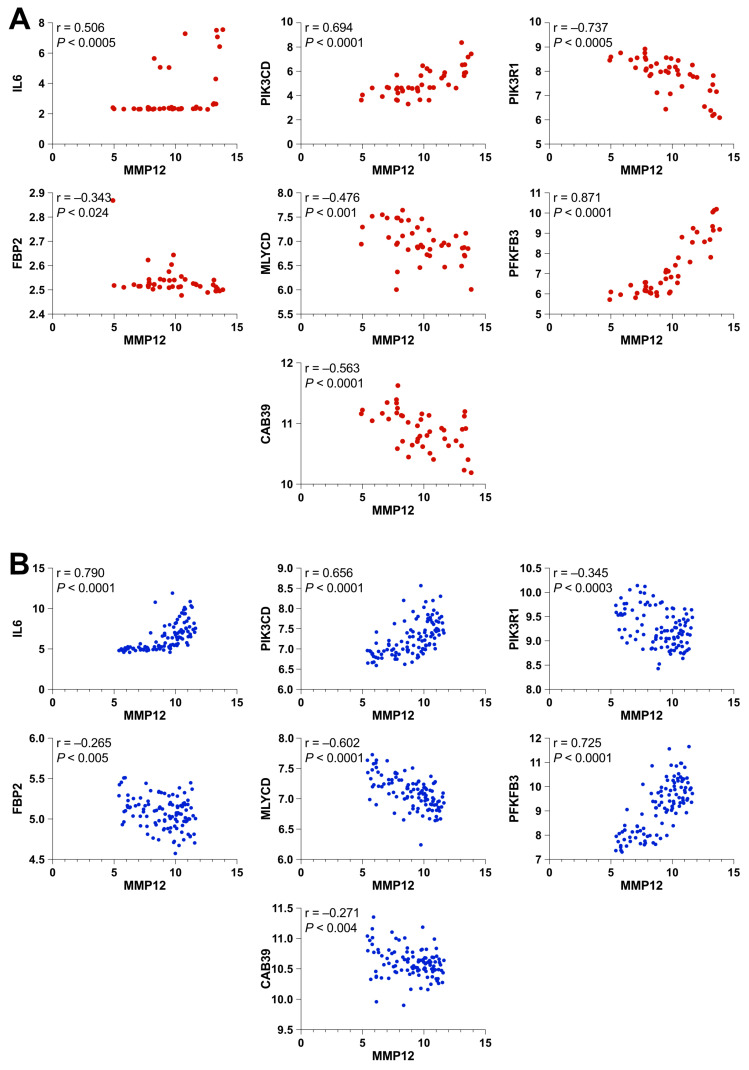
*MMP12* expression correlates with an AMP-activated protein kinase (AMPK)/ mechanistic target of rapamycin (mTOR) transcriptional signature in IBD patients. Correlations of the indicated transcripts in colon biopsy samples from UC patients from the GEO microarray datasets (**A**) GSE38713 [non-IBD (*n* = 13), UC-inactive (*n* = 8), affected UC-active (*n* = 15), and unaffected UC-active (*n* = 7)] and (**B**) GSE59071 [non-IBD (*n* = 11), UC-inactive (*n* = 23), and UC-active (*n* = 74)]. r = Spearman correlation coefficient; *P* = *p*-value.

To gain mechanistic insights into the signaling pathways involved in the regulation of *MMP12* expression, we performed a correlation analysis in two different microarray datasets of patients with UC treated with conventional therapy and showing different disease severities. We focused on the following particular transcripts: *IL-6* (interleukin-6), *PIK3CD* (phosphoinositide 3-kinase), *PIK3R1* (phosphoinositide-3-kinase regulatory subunit 1), *FBP2* (fructose-1,6-bisphosphatase), *MLYCD* (malonyl-CoA decarboxylase), *PFKFB3* (6-phosphofructo-2-kinase/fructose-2,6-biphosphatase 3), and *CAB39* (calcium binding protein 39), which are related to the AMPK and mTOR signaling pathways and were retrieved from the KEGG database. As shown in Figure 2A, *MMP12* expression was significantly correlated with all aforementioned mTOR-/AMPK-related transcripts in the inactive and active UC patients. This observation was corroborated in an additional cohort of patients with similar characteristics (Figure 2B).

### 2.3. MMP12 Expression Is Regulated by AMPK and mTOR in RAW 264.7 Macrophages

**Figure 3:**
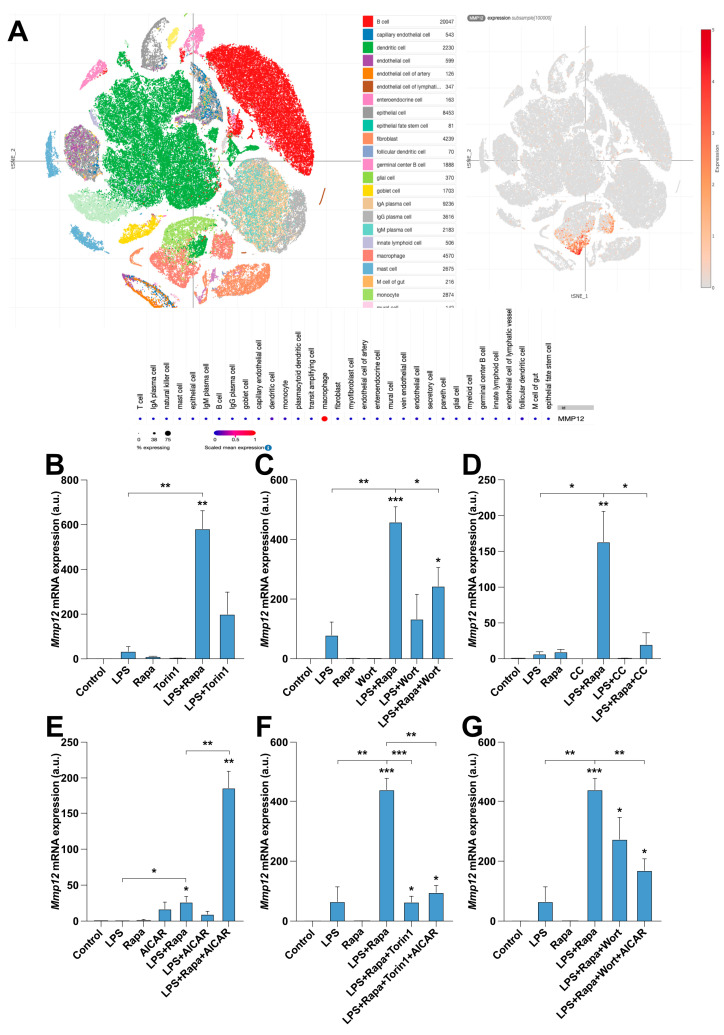
*MMP12* is expressed in macrophages and is regulated by mTOR and AMPK. (**A**) Representative atlas of a CD colon sample containing the indicated plotted cells, the expression levels of *MMP12,* and a summary of the plotted cells retrieved from the Broad Institute’s scRNAseq dataset Single-Cell RNAseq PREDICT 2021 paper: CD. (**B**–**G**) Relative mRNA expression of *MMP12* under the indicated treatments. The data shown in panels (**B**–**G**) represent the mean ± SEM. *, *p* ≤ 0.05; **, *p* ≤ 0.01; ***, *p* ≤ 0.001 relative to the control, unless indicated differently. *n* = at least 3 independent experiments, with 2 biological replicates per sample.

We confirmed that macrophages were the primary *MMP12*-expressing cells in the context of intestinal inflammation. We analyzed the data from a single-cell RNAseq study reported in the PREDICT 2021 paper; CD was retrieved from the Single Cell Portal (Broad Institute). Indeed, among the wide variety of cells in the colon mucosa, macrophages were the main *MMP12*-producing cells (Figure 3A). This observation led us to investigate the former association between AMPK/mTOR pathways and this metalloproteinase in the mouse macrophage cell line RAW 264.7. The selection of this cell line was based on its stability and reproducibility compared to those of human cell lines and the lack of possible ethical issues derived from drawing blood from healthy subjects. Also, RAW 264.7 cells maintain a robust and well-known inflammatory response, especially when challenged with LPS.

The addition of the mTORC1 inhibitor rapamycin clearly caused a synergistic upregulation of *Mmp12* expression when combined with a classic macrophage activator such as LPS, whereas the dual mTORC1/mTORC2 inhibitor Torin1 slightly increased the mRNA levels of that metalloproteinase under the same conditions (Figure 3B). This initial finding suggested that, although mTORC1 blocked the induction of *Mmp12*, other signaling routes should be considered equally relevant. In fact, the addition of the PI3K/AKT inhibitor wortmannin abolished part of rapamycin’s effect on *Mmp12* expression (Figure 3C). Similarly, an AMPK blockade using compound C exerted a drastic inhibition of the synergistic effect of LPS plus rapamycin (Figure 3D), demonstrating that mTORC1 requires the participation of AKT and AMPK. This was further corroborated by stimulating RAW 264.7 macrophages with the AMPK agonist AICAR, which, as opposed to compound C, significantly potentiated the effect of rapamycin plus LPS on *Mmp12* expression (Figure 3E). For completeness, we also wanted to test whether the inhibition of both mTOR complexes or PI3K/AKT affected the transcriptional regulation of *Mmp12* under these conditions of AMPK hyperactivation. As shown in Figure 3F,G, Torin1 or wortmannin treatment significantly decreased the synergistic effects triggered by AICAR.

### 2.4. Glycolysis Regulates MMP12 Expression in RAW 264.7 Cells

**Figure 4:**
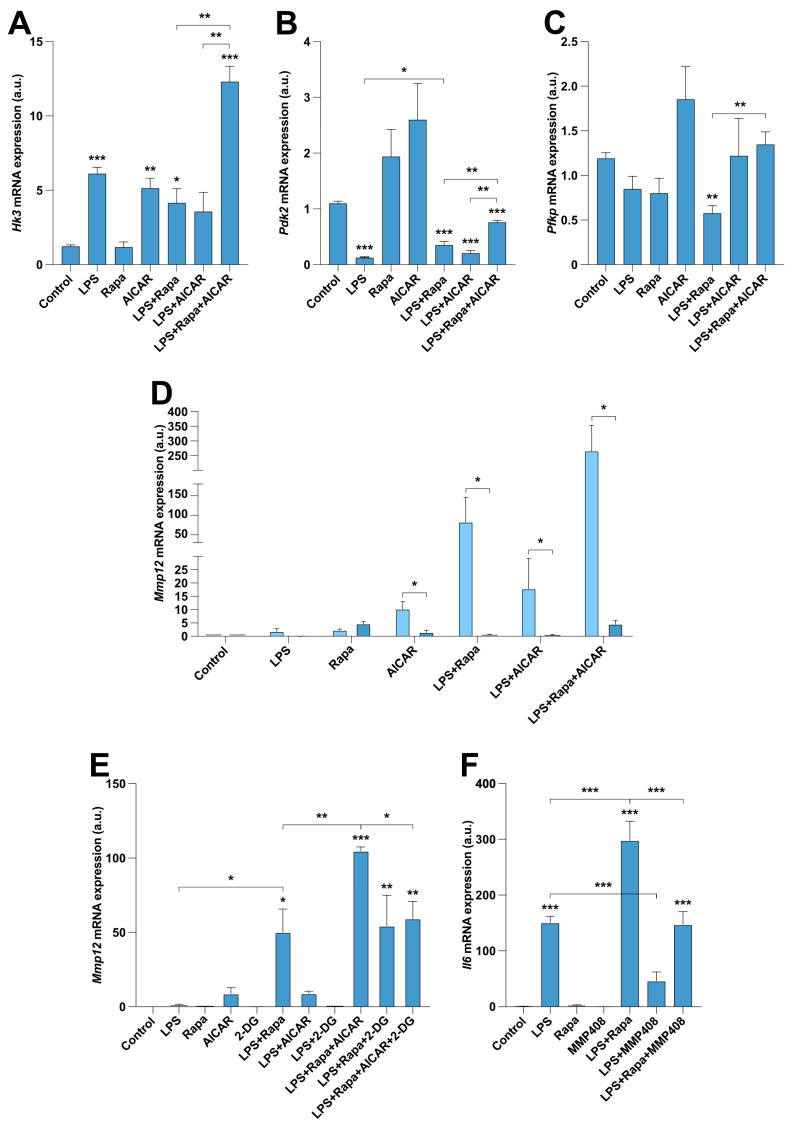
Glycolysis is critical for the induction of *MMP12* in macrophages. (**A**–**F**) Relative mRNA expression of the investigated transcripts under the indicated treatments. The data shown in panels (**A**–**F**) represent the mean ± SEM. *, *p* ≤ 0.05; **, *p* ≤ 0.01; ***, *p* ≤ 0.001 relative to the control, unless indicated differently. *n* = at least 3 independent experiments, with 2 biological replicates per sample.

AMPK and mTOR function as metabolic sensors, and it has been established that activated macrophages undergo a metabolic switch from oxidative phosphorylation to glycolysis to meet the specific requirements in highly energy-demanding conditions, which is not observed in resting monocytes. Therefore, we explored whether this mTOR–AMPK–glycolysis axis could be involved in the regulation of *Mmp12* expression under the promotion of an active and highly glycolytic state by LPS. As a first approach, we analyzed the expression of relevant genes in the glycolysis pathway. We observed that rapamycin only partly blocked the effects of LPS on the expression of *Pdk2* (Figure 4B). However, AICAR was able to increase the expression of *Hk3*, *Pdk2*, and *Pfkp* when added alone or in combination with LPS plus rapamycin (Figure 4A–C), which matched the above-described highest *Mmp12* induction exerted by this AMPK agonist. To confirm the participation of glycolysis in the mechanisms regulating *Mmp12* expression, we cultured RAW 264.7 macrophages in the absence of glucose. As shown in Figure 4D, cells incubated without glucose presented a clear decrease in *Mmp12* transcripts, especially those treated with AICAR (alone or in combination with LPS or LPS plus rapamycin) and the combination of LPS plus rapamycin. In line with these experiments, the addition of the synthetic D-glucose analog 2-deoxy-D-glucose (2-DG, which acts as a competitive inhibitor of glucose metabolism, inhibiting glycolysis via its actions on hexokinase) also abolished the synergism produced by AICAR when combined with LPS and rapamycin (Figure 4E).

### 2.5. MMP12 Inhibition Reduces IL6 Expression in RAW 264.7 Macrophages

Finally, we determined the functional implications of blocking MMP12 in RAW 264.7 mouse macrophages. We analyzed the expression of the essential macrophage activation marker and pro-inflammatory mediator Il6. We found an *Il6* expression pattern that was similar to that observed for *Mmp12*, with a synergistic induction of *Il6* in cells treated with LPS plus rapamycin compared with that in cells stimulated with LPS alone (Figure 4F). Interestingly, the addition of the MMP12 inhibitor MMP408 resulted in a significant decrease in *Il6* expression induced by LPS or the combination of LPS and rapamycin (Figure 4F), corroborating the main role of MMP12 in macrophage biology.

## 3. Discussion

In this study, we corroborated and expanded the data demonstrating the association between MMP12 and IBD. Due to the restricted MMP12 expression in macrophages, we investigated its regulation in those phagocytes. Mechanistically, we described the essential role played by the metabolic mTOR–AMPK–glycolysis axis in the control of *MMP12* expression for the first time. Particularly, mTORC1 inhibition, together with exacerbated AMPK activation and functional glycolysis, drives *Mmp12* overexpression.

Although fecal calprotectin is widely used in clinical practice to diagnose and monitor disease activity [23,24], more biomarkers are still needed to cover calprotectin´s limitations. Its sensitivity and specificity decrease in children and in patients with no inflammatory affectation of the large intestine [25,26]. Therefore, isolating additional stool markers that are complementary to calprotectin is currently an objective for many researchers. In one scenario, stool MMP12 significantly discriminated pediatric UC and CD patients from healthy individuals [22]. Similarly, our results, along with previous observations [18,19,27], also confirmed the elevated expression of *MMP12* in the intestinal mucosa of adult patients with UC and CD. Likewise, we demonstrated that the colonic expression of this metalloproteinase was strongly associated with the activity of the disease and the response to biological therapies. Therefore, MMP12 merits further research to corroborate its utility in IBD monitoring, especially when other members of this family of proteins, such as MMP9, show high accuracy in detecting active UC [28]. It is also worth noting that, to our knowledge, the data presented in previous sections confirm and expand the strong association between the colonic expression of *MMP12* and the responsiveness to biological treatments [29]. This is interesting because it has been demonstrated that MMP12 is able to cleave different anti-TNF antibodies [30], which might explain the increased *MMP12* levels in anti-TNF non-responder patients. However, the fact that vedolizumab-treated patients presented a similar expression pattern makes us think that, most likely, *MMP12* expression is a surrogate of the inflammatory status rather than a marker of therapeutic response. Future analysis of colon biopsies from patients treated with additional biologicals or small molecules will be necessary for further discussion.

Metalloproteinases are no longer considered mere ECM regulators, and their participation in many other processes leading to disease has been widely demonstrated [3,31]. For instance, it was recently shown that some of these proteins were key genes involved in colitis and cancer-associated colitis development [32]. Accordingly, the understanding of MMPs´ adequate control is of the utmost importance. However, little is known about the molecular routes activating *MMP12* transcription in macrophages. Our first approach was intended to correlate the transcript levels of *MMP12* with the central metabolic pathways, such as those of AMPK, mTOR, and glycolysis. The positive correlation between *MMP12* and *PFKFB3* is not surprising, since this factor is also positively associated with AMPK and glycolysis [33]. On the other hand, the PI3K subunit p85 encoded by the gene *PIK3R1* antagonizes the actions of the p110 subunit (encoded by *PIK3CD*) [34], which might explain the observed indirect and direct correlations, respectively. The negative correlation of *MMP12* with *FBP2* could be explained by the opposite effect of this factor with the glycolytic enzyme phosphofructokinase in the promotion of glycolytic flux [35]. Similarly, *CAB39* is functionally associated with the activation of oxidative phosphorylation [36]. Anyway, as far as we know, our functional experiments clearly demonstrated for the first time that combining an mTORC1 blockade with AMPK signaling stimulation in activated macrophages resulted in a dramatic increase in the mRNA expression of *MMP12*. mTOR is vital for macrophage function and activation [37]. Indeed, the overactivation of this serine/threonine kinase increases the production of several pro-inflammatory cytokines [38] and heightens the inflammatory response in bone-marrow-derived macrophages (BMDMs) [39]. Also, intestinal lamina propria macrophages lacking the expression of *Il10* showed increased mTORC1 activity, mitochondrial reactive oxygen species (ROS) production, and inflammasome activation, and this might have contributed to the development of colitis in *Il10*-deficient mice [40]. It is, therefore, not surprising that the modulation of mTOR functions affects MMP levels. Nevertheless, opposite to that which was commonly observed for cytokines and chemokines [41,42,43], the inhibition of mTOR signaling tended to upregulate the expression of MMPs. In fact, it was recently described that the attenuation of mTOR in THP-1 macrophages by rapamycin or silica dust induced the expression of relevant metalloproteinases, such as MMP1 and MMP9 [44,45]. Consistently with this, the exacerbation in the induction of a well-defined detrimental factor such as MMP12 by the activation of AMPK is rather unexpected, since the upregulation of this pathway typically counteracts excessive inflammatory responses in macrophages by mediating the effects of anti-inflammatory cytokines or inhibiting the production of pro-inflammatory mediators such as TNF-α and IL6 [46,47,48]. Similarly, specific knockout of the AMPK subunit β1 in macrophages exacerbated experimental colitis symptoms [49]. However, contradictory results regarding AMPK’s modulation of MMP expression have been published. *AMPKα1* knockout exacerbated the expression of MMP14 in mouse BMDMs treated with LPS [50]. By contrast, the AMPK inhibitor compound C inhibited the induction of MM9 and MMP13 elicited by PMA in THP-1 macrophages [51], suggesting that AMPK can either stimulate or block MMP production depending on the initial trigger and the downstream cellular routes involved. In fact, our results showed a strong dependency of *Mmp12* expression on functional glycolysis, especially when RAW264.7 cells were treated with AICAR. In agreement with this, the addition of the glycolytic inhibitor 2-DG to chondrocytes repressed the expression of *MMP13* [52] and B-cell adapter for PI3K (*BCAP*) knockout BMDMs, which showed impaired glycolysis and expressed less MMP9 after LPS treatment [53], thereby highlighting the influence of this metabolic route on the transcription of specific MMPs.

Finally, given the observed transcriptional correlation between *MMP12* and the classic proinflammatory cytokine *IL6* in the colon mucosa of IBD patients (Figure 2), we wanted to test whether macrophages could contribute to that association. Indeed, the inhibition of mTOR signaling also synergized with LPS in the induction of *Il6* transcripts. Consistently with knocking down the expression of *Mmp12* [54], adding MMP408 decreased the mRNA expression of *Il6* elicited by LPS in RAW 267.4 macrophages. Interestingly, we noted that all of the synergism caused by the supplementation with rapamycin was abolished by the inhibition of MMP12, demonstrating that this metalloproteinase is crucial for the transcriptional regulation of *Il6*, especially when mTORC1 signaling is impaired. It is noteworthy that Il6 was able to increase the expression of Mmp12 in mouse macrophages [55], which might support the existence of a feedback loop between these two factors in macrophages.

**Figure 5:**
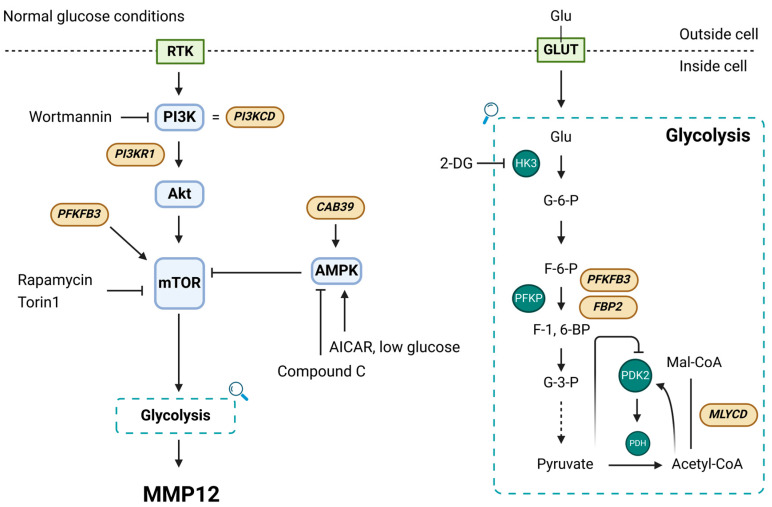
Summary of the mechanisms involved in the regulation of MM12 transcription.

In summary, our study demonstrated a strong association between MMP12 and the pathology of IBD. Moreover, the opposing AMPK and mTOR pathways [56], together with functional glycolysis, play a significant role in the induction of this metalloproteinase in macrophages. Our data also showed how Mmp12 engages with the transcription of the proinflammatory cytokine *Il6*, suggesting the participation of Mmp12 in functions beyond ECM remodeling, such as the activation of these phagocytes. Future studies will unveil the relevance of the mTOR/AMPK–glycolysis–MMP12 axis in the development and progression of immune-mediated diseases such as IBD (Figure 5). There is an obvious limitation of this study, which is the lack of corroboration of our results in terms of protein expression. Our initial hypothesis was indeed focused on transcriptomic data, and we followed that rationale. However, we also consider that putative differences between mRNA and protein expression should be taken into account.

A schematic representation of the putative interactions among all factors analyzed in the current study is provided. It should be noted that all genes in brown boxes are located in a diagram based on the current literature, and these results were not demonstrated in this work (created with BioRender.com).

## 4. Materials and Methods

### 4.1. Bioinformatics of Human Transcriptomic Datasets

Statistical analyses and text processing were carried out using the R (version 3.3.1) and Perl software version 5.38.2, respectively. For the microarray analysis, signal intensity values were obtained from CEL files after a robust multichip average [57,58]. Differentially expressed genes were identified using linear models for microarray data [59]. Adjusted *p*-values for multiple comparisons were calculated by applying the Benjamini–Hochberg correction method [60]. Volcano plots were generated with Glimma (https://doi.org/10.1093/nargab/lqab116, accessed on 10 August 2022) by plotting the log2 fold changes and corresponding −log10 *p*-values obtained from the differential expression analyses performed with limma (linear models for microarray data) [59]. All microarray expression datasets were obtained from the Gene Expression Omnibus (GEO) database (http://www.ncbi.nlm.nih.gov/geo/, accessed on 21 February 2023). The accession codes for the datasets used in this work were the following: (i) GSE38713 [61] (diagnosis of UC at least 6 months before inclusion, “active” was defined with an endoscopic Mayo subscore of >2, “inactive” defined as Mayo subscore = 0, “uninvolved” was defined as a colonic segment with a completely normal appearance and histology and no previous signs of active disease) and GSE59071 [62] (activity was endoscopically assessed) for colon biopsies from UC patients after treatment with immunosuppressants and/or corticosteroids; (ii) GSE16879 [63] for colon biopsies from UC, CD, and non-IBD controls, as well as UC patients treated with anti-TNF therapy (response was defined based on endoscopic and histologic findings at 4–6 weeks); (iii) GSE73661 [64] for colon biopsies from UC and non-IBD controls, as well as UC patients treated with anti-TNF and vedolizumab therapy (response was defined as endoscopic mucosal healing with a Mayo endoscopic subscore of 0 or 1 at weeks 4–6 for anti-TNF and 6–12–52 for VDZ); (iv) GSE23597 [65] for colon biopsies from moderate to severe UC patients at baseline, 8 weeks, and 30 weeks after anti-TNF therapy. Also, scRNAseq data from the Broad Institute dataset in the Single Cell RNAseq PREDICT 2021 paper (CD) were used (https://singlecell.broadinstitute.org/single_cell, accessed on 11 April 2023).

### 4.2. Cell Culture and Treatments

**Figure 6:**
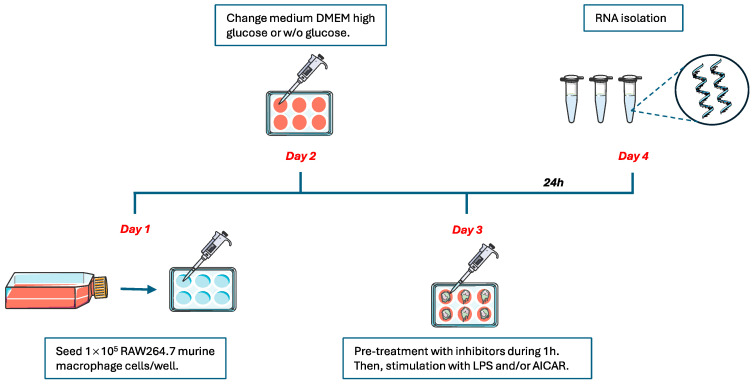
Summary of the experimental design for the cell culture.

RAW 264.7 murine macrophage cells (essentially programmed to M1 macrophages) obtained from the American Type Culture Collection (ATCC, Manassas, VA, USA; Cat. no. TIB-71) were cultured in Dulbecco´s modified Eagle’s medium (DMEM) with high glucose (Lonza, Basel, Switzerland; Cat. no. BE-12-614F) or with no glucose (Gibco, Billings, MT, USA; Cat. no. 12307263) supplemented with 10% fetal bovine serum (Corning Inc., Corning, NY, USA; Cat. no. 35-079-CV), L-glutamine (2 mM) (Lonza, Basel, Switzerland; Cat. no. BE17-605E), and penicillin (50 units/mL)/streptomycin (50 μg/mL) (Corning Inc., Corning, New York, NY, USA; Cat. no. 5530-002-CI) and incubated at 37 °C with 5% CO_2_. For the experiments, cells were seeded in six-well plates at a density of 1 × 10^5^ cells/well. Cells were treated with LPS O26:B6 (100 ng/mL) (Sigma-Aldrich, St. Louis, MO, USA; Cat. no. L2654) or the AMPK agonist AICAR (1 mM) (Sigma-Aldrich, St. Louis, MO, USA; Cat. no. A9978). The specific pharmacological inhibitors rapamycin (10 ng/mL) (Sigma-Aldrich, St. Louis, MO, USA; Cat. no. 553211) (mTORC1), Torin1 (10 ng/mL) (Sigma-Aldrich, St. Louis, MO, USA; Cat. no. 475991) (mTORC1/mTORC2), wortmannin (10 ng/mL) (Sigma-Aldrich, St. Louis, MO, USA; Cat. no. W1628) (PI3K/Akt), Compound C (5 ng/mL) (Sigma-Aldrich, St. Louis, MO, USA; Cat. no.171260) (AMPK), 2-DG (0.5 mM) (Sigma-Aldrich, St. Louis, MO, USA; Cat. no. D8375) (glycolysis), and MMP408 (100 ng/mL) (Sigma-Aldrich, St. Louis, MO, USA; Cat. no. 444291) (MMP12) were added 1 h before stimulation with LPS. Cells remained in culture for 24 h before isolation (Figure 6).

### 4.3. RNA Isolation and Real-Time Reverse Transcription–Polymerase Chain Reaction (RT-qPCR)

The total RNA from RAW 264.7 cells was extracted with NZYol (NZYTech, Lisboa, Portugal; Cat.no. MB18501) and the E.Z.N.A Total RNA Kit (Omega Bio-tek, Norcross, GA, USA; Cat. no. R6834-02) according to the manufacturer’s instructions. The quantity and quality of the total RNA were assessed using the NanoDrop ND-1000 spectrophotometer (NanoDrop Technologies, Wilmington, DE, USA). Reverse transcription–quantitative real-time polymerase chain reaction (RT-qPCR) was performed using a Power SYBR™ Green RNA-to-CT™ 1-Step Kit (Applied Biosystems, Waltham, MA, USA; Cat. No. 4389986) by using 10 ng of total RNA per sample. The data were calculated using the comparative (2^−ΔΔCt^) method and Quantstudio Design and Analysis Software v.1.5.3 (Applied Biosystems, Waltham, MA, USA). Control cells were used as a reference and were assigned an arbitrary number of 1; we established that the higher Ct range was at 35. The specific PCR primers used are included in Table 1 (Thermo Fisher Scientific, Waltham, MA, USA), and *Il-6* (NM_031168.2) (Sigma-Aldrich, St. Louis, MO, USA; Cat. no. KSPQ12012) was also used. Note that the gene expression levels were normalized to that of *β-actin.* Melting curves were generated to ensure a single gene-specific peak, and no-template controls were included for each run and each set of primers to control for unspecific amplifications.

### 4.4. Statistical Analysis

Statistical analysis was performed by using the GraphPad Prism 8.0.1 software (GraphPad Software, Boston, MA, USA). Quantitative data are presented as the mean ± standard error of the mean (SEM) of at least three independent experiments and are represented as the fold-change versus controls. Two-group comparisons were performed using a two-tailed Student *t*-test. Multigroup comparisons were performed using one-way ANOVA followed by the Bonferroni multiple-comparison test after the application of the appropriate normality test. A *p*-value of <0.05 was considered statistically significant. A Spearman correlation test of the investigated genes (shown in Figure 2) was performed using the GraphPad Prism 8.0.1 software (GraphPad Software, Boston, MA, USA) and the normalized data of the microarray datasets indicated in the appropriate figure.

A schematic representation of the protocol used for in vitro experiments with RAW 264.7 cells is shown in the figure.

## Figures and Tables

**Table 1:** List of primers used for RT-qPCR.

Gene	Forward	Reverse
*Mmp12*	TTAAGGGAACTTGCAGTCGG	TCTTGACAAGTACCATTCAGCA
*Hk3*	TTTCGGTTAAGTGGCTACAGAGG	TTGCTGCAAGCATTCCAGTT
*Pdk2*	GCGCTGTTGAAGAATGCGT	CCTGCCGGAGGAAAGTGAAT
*Pfkp*	GGGCAGACACAGCTCTGAAC	CACTCCTTTGCCCTCCTCTG
*β-actin*	CAGCTTCTTTGCAGCTCCTTC	ACCCATTCCCACCATCACAC

## Data Availability

The data are available upon request to the corresponding author.
